# Complementary and alternative medicine use among women at increased genetic risk of breast and ovarian cancer

**DOI:** 10.1186/1472-6882-8-17

**Published:** 2008-04-30

**Authors:** Christine M Mueller, Phuong L Mai, Jaime Bucher, June A Peters, Jennifer T Loud, Mark H Greene

**Affiliations:** 1 Clinical Genetics Branch, Division of Cancer Epidemiology and Genetics, National Cancer Institute, National Institutes of Health, Bethesda, Maryland, USA; 2 College of Medicine, University of Toledo, Toledo, Ohio, USA

## Abstract

**Background:**

Complementary and alternative medicine (CAM) use is well documented among breast cancer patients and survivors, but little evidence is available to describe rates and patterns of use among women at increased genetic risk of breast cancer.

**Methods:**

A pre-visit telephone interview was conducted to ascertain CAM use among the *BRCA *mutation carriers enrolled in a high-risk breast cancer screening study. Participants were asked to report on their use of thirteen therapies within the year prior to enrollment into the study. Logistic regression was used to evaluate the association between various factors and CAM use in this population.

**Results:**

Among the 164 *BRCA1 *or *BRCA2 *mutation-positive (*BRCA*+) women in this analysis, 78% reported CAM use, with prayer and lifestyle diet being the two most commonly reported modalities. Many subjects used multiple CAM therapies, with 34% reporting use of three or more modalities. The most commonly used modalities were mind-body therapies and biologically-based practices, 61.6% and 51.8%, respectively. High-risk women were more likely to use CAM if they were older, more educated, more worried about ovarian cancer risk, or had a previous cancer diagnosis.

**Conclusion:**

This study suggests that the prevalence of CAM use is high among *BRCA *mutation carriers, with frequency of use comparable to that of breast cancer patients and survivors. Given the high prevalence of CAM use in our subjects, especially biologically-based therapies including herbal supplements, whose safety and efficacy in relation to cancer risk are unknown, our study suggests that future research is necessary to clarify these risks, and that it is important for providers to inquire about and to discuss the pros and cons of CAM use with their *BRCA+ *patients.

## Background

Complementary and alternative medicine (CAM) includes a collection of therapies, practices, and products that are not considered to be part of conventional medical practice [1]. The National Center for Complementary and Alternative Medicine (NCCAM) divides CAM therapies into five domains, recognizing that overlap exists. They include (with examples): biologically-based practices (herbs, diets), mind-body medicine (prayer, meditation, yoga), energy healing (Reiki, therapeutic touch), manipulative and body based practices (chiropractic care, massage), and whole medical systems (traditional Chinese medicine, Ayurveda). Use of CAM escalated in the United States during the 1990's, with an estimated $36 to $47 billion spent on these modalities in 1997 alone. In addition, several studies have shown that CAM is most commonly used in conjunction with conventional medical interventions [2,3].

The 2002 National Health Interview Survey (NHIS) of 31,044 US adults, of whom more than 17,000 were women, provides the most recent CAM use data for the general population. In that survey, CAM use was greater among women than men, with sixty-nine percent of women reporting CAM use within the 12 months prior to survey completion. Prayer was the most commonly used modality. When prayer for health reasons was excluded, the prevalence of CAM use among women decreased to 40%. Higher levels of CAM use were associated with increased education, higher income, and poorer health status. In general, women between 50–59 years of age comprised the sub-group with the highest rates of use for most CAM modalities, except for prayer, which continued to increase with advancing age [2,4].

Several studies have shown that cancer patients and survivors are more likely to use CAM than unaffected individuals in the general population, with up to 83% reporting CAM use [5-8]. Furthermore, women with a current or previous diagnosis of breast cancer have been reported to use CAM more frequently than individuals diagnosed with other cancers [9]. Self-reported motivations for CAM use among recently-diagnosed patients include desires to improve quality of life, enhance immune function, and actively participate in self-care [6,10]; higher levels of anxiety and depression have been associated with higher rates of CAM use among cancer patients and survivors [11-13].

While there is an extensive literature regarding CAM use among breast cancer patients, much less is known about its use among women at high genetic risk of developing breast cancer. In a previous study of CAM use within a cohort of women undergoing genetic counseling and testing for germline mutations in *BRCA1/2 *genes, cancer survivors reported significantly more CAM use than unaffected women [14]. A follow-up to that study showed that *BRCA+ *women were more likely to be using CAM one year after disclosure of their genetic test result, compared with usage rates prior to genetic testing [15].

Women at increased genetic risk of breast and ovarian cancer face difficult decisions when choosing the optimal strategy to manage their cancer risk. Their decisions are often based on personal beliefs, attitudes, and experiences, as expressed by their cancer risk perception and worry [16-20]. Furthermore, high-risk women who choose salpingo-oophorectomy as an ovarian cancer risk-reducing option must also face medical decisions regarding the management of postmenopausal symptoms related to surgical menopause. For the general population and breast cancer survivors, this often includes taking herbal supplements, such as soy products, whose safety and efficacy are unproven [21,22]. With the increased prevalence of CAM usage in the general population, it is important to identify patterns of use among high-risk women to promote informed medical decision-making and to improve the quality of care that these patients receive. In this study, we comprehensively described and analyzed CAM use in women with known *BRCA1 *or *BRCA2 *mutations enrolled in a high-risk breast cancer screening study.

## Methods

The Clinical Genetics Branch Breast Imaging Screening Study in Women at High Genetic Risk of Breast Cancer: Annual Follow-up Study (NCI protocol 01-C-0009) is aimed at evaluating breast cancer screening modalities among women who are genetically predisposed to developing breast cancer. The study consists of four consecutive annual evaluations including mammogram, breast MRI, clinical breast examination, transvaginal ultrasound, CA-125 and breast ductal lavage. As part of this protocol, participants were asked to complete a self-administered questionnaire and a telephone questionnaire prior to their first study visit. Participants were eligible for the study if they were between 25 and 56 years of age, had a known *BRCA1 *or *BRCA2 *mutation, or were first- or second-degree relatives of a known *BRCA *mutation-positive family member. Women younger than 25, but within 5 years of the age at diagnosis of the youngest family member with a hereditary breast-ovarian cancer syndrome-associated tumor, were also eligible.

Exclusion criteria included pregnancy or lactation within 6 months of enrollment, abnormal CA-125 level, bilateral breast cancer, previous bilateral mastectomy or bilateral radiation therapy, weight over 136 kg, or allergy to gadolinium. Individuals with a personal history of ductal carcinoma *in situ *(DCIS), Stage I, or Stage IIA breast cancer were eligible provided that at least 6 months had elapsed since completing primary therapy (surgery, radiation, or chemotherapy). Other exclusion criteria included a personal history of ovarian cancer (any stage); breast cancer (stage IIB or higher); DCIS, Stage I or Stage IIA breast cancer with a relapse after primary treatment; or any other invasive cancer except non-melanoma skin cancer or cervical carcinoma *in situ*, unless relapse-free for 5 years prior to the time of enrollment. Informed consent was obtained from all participants, and a total of 200 women were enrolled onto 01-C-0009 between June 2002 and February 2007. A secondary study objective of the study was to determine the rates of CAM use in high-risk women enrolled in this breast cancer screening study related to various participant characteristics and screening behaviors. Of the participants in the Breast Imaging Screening Study, 164 women who know their *BRCA+ *status and completed their CAM questionnaire were included in this analysis.

### Demographics

Demographic information, including age, race (white, non-white), marital status (single, married/steady relationship, separated/divorced), number of children (0, ≥1), education (high school or less, college and beyond), and personal cancer history, was obtained through the pre-visit questionnaires.

### Complementary and Alternative Medicine Use

Complementary and alternative medicine data were obtained through the pre-visit telephone interview, which was completed prior to the first study visit. Participants were asked to answer "yes" or "no" to the use of each of thirteen CAM therapies (acupuncture, meditation, relaxation techniques, yoga, massage therapy, imagery, spiritual healing or prayer, lifestyle diet, herbal medicine, homeopathic treatment, energy healing, biofeedback, and hypnosis) within the previous year. No standard examples of these modalities were provided by the interviewer, and no further details regarding frequency, duration, or purpose of use were elicited. Participants were also given the opportunity to report the use of additional therapies. For data analysis purposes, the individual CAM therapies were further grouped by the defined NCCAM domains: biologically-based practices (herbal medicine, lifestyle diet); mind-body medicine (spiritual healing or prayer, meditation, yoga or tai chi, relaxation techniques, biofeedback, imagery, hypnosis); energy healing, manipulative and body-based practices (massage therapy); and alternative medical systems (homeopathic treatment, acupuncture).

### Perceived Cancer Risk

Perceived breast and ovarian cancer risk was assessed for each cancer with the survey question: "In your opinion, compared to other women your age, what are your chances of getting *breast/ovarian *cancer in your lifetime?" Participants responded with categorical options: "Much less," "A little less," "About the same," "A little more" or "Much more," coded on a 1–5 scale. For the current analysis, responses were dichotomized to average or below average (codes 1–3) and above average (codes 4,5) risk perceptions [16]. Individuals who chose not to answer the question were excluded from the cancer risk perception analysis.

### Cancer Worry

Cancer worry was determined using three validated questions from the Lerman Cancer Worry Scale [23,24], relating to frequency of thoughts of getting either breast or ovarian cancer and their impact on mood and daily activities: "During the past month, how often have you thought about your own chances of getting *breast/ovarian *cancer; how often have thoughts about your chances of getting *breast/ovarian *cancer affected your mood; how often have thoughts about *breast/ovarian *cancer affected your ability to perform your daily activities?" Participants responded with categorical options: "Not at all or Rarely," "Sometimes," "Often," "A lot," coded on a 1–4 scale. The average score was calculated for each cancer to determine their level of cancer worry. Individuals who chose not to answer the questions were excluded from the cancer worry analysis.

### Depression

Depression was measured using the 20 question Center for Epidemiologic Studies Depression (CES-D) Scale, a short self-report instrument designed to measure depressive symptoms in the general population, emphasizing depressed mood, the major affective component of depression [25]. Each question was scored from 0 to 3 based on the frequency of occurrence within the past week, with responses then summed, for a total possible score ranging from 0 to 60. We grouped the scores into <16 and ≥ 16, since a score of ≥ 16 is indicative of clinical depression [25]. For individuals with four or fewer missing responses, a person-mean imputation technique based on the existing responses was utilized to develop a total score. The mean value of the individual's completed items is substituted into the missing items to calculate a total CES-D score as previously described [26]. Individuals with >4 missing responses were excluded from the depression analysis.

### Screening Behavior

Participants provided detailed information on how frequently they performed cancer screening activities before study enrollment. For mammography and transvaginal ultrasound (TVU), we dichotomized participants as either "following" or "not following" appropriate screening guidelines (monthly breast self exam (BSE), annual mammography, annual TVU) based on self-reported screening frequency [16,27]. The date of mutation status disclosure was defined as the time from which appropriate screening practices should have been followed. Individuals who screened more frequently than recommended guidelines were considered to be following guidelines. Women who reported having bilateral oophorectomy (n = 69) were excluded from the TVU data analysis.

### Statistical Analysis

For comparison between CAM users and non-users, Chi-square and Fisher's Exact tests were used as appropriate for categorical variables, and Wilcoxon rank-sum test was used for continuous variables. We used logistic regression to evaluate the association between various factors and CAM use. Factors with a p-value of ≤ 0.20 in the univariate analysis were included in the final multivariate logistic regression model. Factors with a two-tailed p-value of ≤0.05 in the multivariate model were considered to be statistically significant. All statistical analyses were conducted using SPSS 15.0.

## Results

Of 165 *BRCA+ *women who knew their mutation status prior to enrolling in the Breast Imaging Screening Study, 164 answered the pre-visit telephone CAM questionnaire and were included in this analysis. The cohort was highly-educated (92.7% attending college); predominately white (97.6%); most were married (72%); over half reported having children (54.3%); and 17.7% had a personal history of cancer prior to study entry.

**Figure 1:**
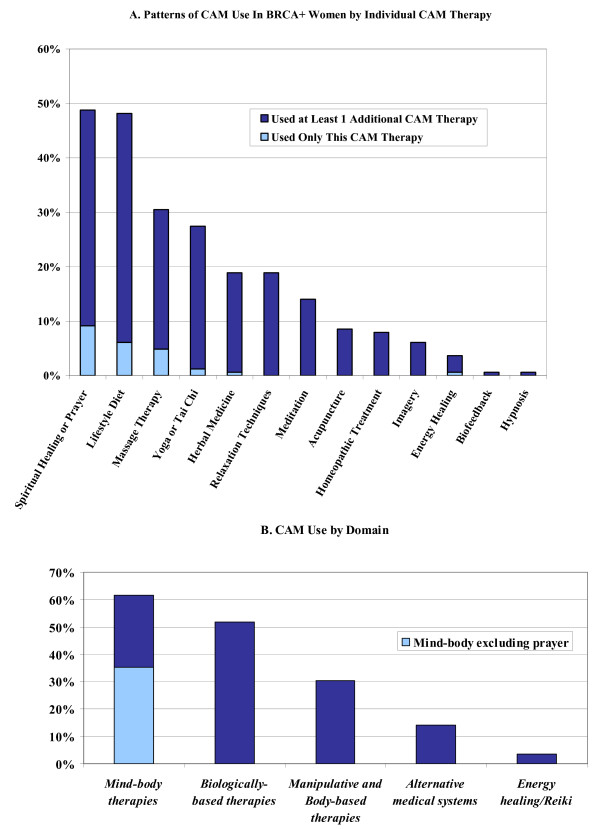
Patterns of CAM Use in BRCA+ Women.

The overall CAM use rate in our *BRCA*+ population was 78% (128/164), with an average of 2.3 CAM therapies used per person. Thirty-four percent of the cohort reported having used three or more CAM therapies within the past year. As shown in Figure 1A, only 15 (9.1%) *BRCA*+ women reported prayer as the sole CAM therapy used. If spiritual healing/prayer are excluded, 68.9% of participants had used CAM in the previous year. Spiritual healing/prayer and lifestyle diet were the most commonly reported modalities (48.8% and 48.2%, respectively). Figure 1B shows CAM use grouped by NCCAM domains: biologically-based practices (herbal medicine, lifestyle diet); mind-body medicine (spiritual healing or prayer, meditation, yoga or tai chi, relaxation techniques, biofeedback, imagery, hypnosis); energy healing, manipulative and body-based practices (massage therapy); and alternative medical systems (homeopathic treatment, acupuncture). Mind-body therapies and biologically-based practices were the most commonly used domains (61.6% and 51.8%, respectively). However, if prayer is excluded from the computations, biologically-based practices become the most prevalent modality.

There were significant positive associations between CAM use and several demographic, health-related, and psychosocial variables (Tables 1 and 2). Higher education (p = 0.015), a previous cancer diagnosis (p = 0.006), and older age (p = 0.007) were all associated with increased CAM use. Although the overall scores were not high (median = 1.33), higher ovarian cancer worry scores were statistically significantly associated with increased CAM use (p = 0.011) (Table 2). These findings did not change when the ovarian cancer worry analysis was restricted to the 95 women with intact ovaries (data not shown); therefore, all women were included in the multivariate regression analysis. Among the demographic indicators, race (p = 1.00), number of children (p = 0.52), and marital status (p = 0.91) were not associated with CAM use, and neither were the health-related behaviors of breast or ovarian cancer screening, nor the psychosocial variables depression, perceived cancer risk, and breast cancer worry.

**Table 1:** Associations Between CAM Use and Demographic, Psychosocial and Health-related Categorical Predictor Variables in *BRCA+ *Women

**Variable**	**CAM User (%)**	**CAM Nonuser (%)**	**p**
***Education***			
High School or Less	6 (4.7)	6 (16.7)	***0.015***
Some College and Beyond	122 (95.3)	30 (83.3)	
***Race/Ethnicity***			
White	125 (97.7)	35 (97.2)	1.00
Non-White	3 (3.3)	1 (2.8)	
***Children***			
0	56 (43.8)	17 (50.0)	0.52
≥1	72 (56.3)	17 (50.0)	
***Marital Status***			
Single	27 (21.3)	7 (19.4)	0.91
Married/Steady Partner	91 (71.7)	27 (75.0)	
Separated/Divorced	9 (7.1)	2 (5.6)	
Previous Cancer History*			
Yes	28 (21.9)	1 (2.8)	***0.006***
No	100 (78.1)	35 (97.2)	
***CES-D Score***			
Depressed	25 (19.7)	6 (16.7)	0.68
Not Depressed	102 (80.3)	30 (83.3)	
***Breast Cancer Risk Perception***			
Average or Below Average Risk	3 (2.4)	1 (2.9)	1.00
Above Average Risk	121 (97.6)	33 (97.1)	
***Ovarian Cancer Risk Perception***			
Average or Below Average Risk	34 (27.4)	11 (32.4)	0.57
Above Average Risk	90 (72.6)	23 (67.6)	
***Breast Self Exam***			
Less than Once per Month	58 (46.0)	11 (31.4)	0.12
At Least Once per Month	68 (54.0)	24 (68.6)	
***Mammography***			
Not Following Guidelines	25 (19.5)	10 (27.8)	0.29
Following Guidelines	103 (80.5)	26 (72.2)	
Transvaginal Ultrasound *limited to 95 women with ovaries			
Not Following Guidelines	22 (29.3)	4 (20.0)	0.57
Following Guidelines	53 (70.7)	16 (80.0)	

* Number of cancers by site: breast cancer = 23 (two with multiple primaries 1 = melanoma, 1 = basal cell carcinoma), ovarian cancer = 1, melanoma = 1, thyroid = 1, vaginal clear cell adenocarcinoma = 1, basal cell carcinoma = 2

**Table 2:** CAM Use and Demographic, Psychosocial and Health-related Continuous Predictor Variables in *BRCA+ *Women

	**CAM User Median (Range)**	**CAM Nonuser Median (Range)**	**p**
**Age**	41.0 (22.0–55.0)	35.0 (23.0–55.0)	***0.007***
**Breast Cancer Worry**	1.67 (1.00–3.67)	1.33 (1.00–2.67)	0.18
**Ovarian Cancer Worry**	1.33 (1.00–3.67)	1.00 (1.00–2.33)	***0.011***

Factors with p ≤ 0.20 (age, education, previous cancer diagnosis, ovarian cancer worry, breast cancer worry, and BSE) were included in the multivariate logistic regression model. Odds ratios and 95% confidence intervals are presented in Table 3. Age, higher education, previous cancer diagnosis, and increased ovarian cancer worry remained statistically significantly associated with CAM use (p ≤ 0.05). Previous cancer diagnosis showed the strongest association with increased CAM use; women with any previous cancer were 17 times more likely to use CAM than unaffected women. Multivariate analysis showed a weak, yet statistically significant, inverse association between frequency of BSE and CAM use; women who did BSE less than once a month were more likely to use CAM.

**Table 3:** Multivariate Associations Between CAM Use and Predictor Variables in *BRCA*+ Women

	**Odds Ratio (95% CI)**	**p**
**Education**	**10.3 **(2.0–53.8)	***.006***
**Personal Cancer History**	**17.1 **(1.5–191.4)	***.021***
**Breast Self Exam**	**0.3 **(0.1–0.8)	***.017***
**Age**	1.1 (1.0–1.1)	***.016***
**Breast Cancer Worry**	1.0 (0.4–2.4)	.958
**Ovarian Cancer Worry**	**7.9 **(1.7–38.1)	***.009***

Since a previous cancer diagnosis was highly-associated with CAM use in our population, a finding previously-reported in the general population literature, we repeated our analyses after excluding the women with a previous cancer diagnosis. The overall patterns of the CAM therapies used remained unchanged (data not shown), with the exception of lifestyle diet being more commonly used than spiritual healing/prayer (47.4% and 44.4%, respectively) compared with 48.2% and 48.8%, respectively in the previous analysis. When spiritual healing/prayer was excluded, overall CAM use was 65.2% compared to 68.9% when all *BRCA*+ women were included in the analysis.

Age, education, previous cancer diagnosis, ovarian cancer worry, breast cancer worry, and BSE all had p ≤ 0.20 in the univariate analyses again and were included in the multivariate logistic regression model. Ovarian cancer risk perception was also included in the multivariate analyses with p = 0.15. As was the case with the previous analysis, age, higher education, ovarian cancer worry, and BSE remained statistically significantly associated with CAM use (p ≤ 0.05), with similar odds ratios. Ovarian cancer risk perception was no longer statistically significant.

## Discussion

Our study provides the largest, most comprehensive descriptive analysis of CAM use among *BRCA *mutation carriers who are at increased genetic risk of breast and ovarian cancer, and who had undergone risk assessment and counseling prior to enrollment. Compared with data from women in the general population [4], the high-risk women enrolled in our cohort were more likely to use CAM (78% vs. 69%), a rate which is comparable to that reported for breast cancer patients and survivors [5-8]. When prayer is excluded, only 40% of women in the general population used CAM, compared with 68.9% of the *BRCA*+ women in our study [4]. Therefore, although prayer was also the most commonly used CAM modality in our population, in contrast to the pattern of use observed in the general population, it was commonly used in addition to other modalities.

CAM use in our cohort was associated with older age, higher education level, and higher levels of ovarian cancer worry. A previous cancer diagnosis showed the strongest association with increased CAM use in our *BRCA+ *population, consistent with reports based on general population samples [5-8]. In a previous report on high-risk women, cancer survivors reported more CAM use than unaffected women; however, all univariate and multivariate analyses in that study were conducted separately for these groups [14]. Since our sample included only a small number of participants with a previous cancer diagnosis, analyzing CAM use stratified by previous cancer history would have had limited statistical power. As an alternative, we performed our analyses excluding the women with a previous cancer diagnosis, and found no meaningful differences in results.

Previous studies have reported conflicting evidence for the association between age and CAM use both in the general population and in breast cancer patients and survivors [2,6,28,29], while other studies have shown no association between age and CAM use [15,30]. Some of this discordance may be due to the manner in which age is dichotomized in different studies. The age group with the most extensive CAM use in the general population was between 50–59 years [2]. In our study, CAM use increased with age, but the oldest subject in our cohort was only 55. Thus, we were not able to evaluate CAM use in older women.

This study showed no association between CAM use and perceived cancer risk (breast and ovarian) or breast cancer worry. CAM users tended to be more worried about ovarian cancer, but overall the reported levels of breast and ovarian cancer worry were low. Higher rates of CAM use have been associated with increased levels of depression in cancer survivors and individuals with chronic illness in some studies [2,13,31], a finding we did not corroborate. These results may be related to the homogeneity of our study participants, all of whom have been informed of their increased cancer risk through genetic counseling, knew themselves to be *BRCA *mutation-positive; have chosen breast cancer screening rather than prophylactic mastectomy to manage their risk; and, for the majority, have not developed a BRCA-related malignancy. These characteristics, in the aggregate, may yield a group of women which is less worried about developing cancer than the more heterogeneous groups of high-risk women who have been studied previously.

Prior reports have suggested that high-risk women report better health maintenance practices than do women from the general population [14,16]. Therefore, we evaluated potential associations between CAM use and cancer screening practices for breast and ovarian cancer, covariates which have not been previously examined. The majority of our study participants followed recommended screening guidelines. The only significant, although weak, association that we found was that women who did BSE less than once a month were more likely to use CAM.

Limitations to our study include the reliance on self-reported data regarding CAM use. Participants were not provided examples for each of the thirteen modalities, and thus may have interpreted modalities such as lifestyle diet differently. Furthermore, we did not collect data on duration and frequency of use for each CAM, nor did we elicit information regarding specific motives for CAM use such as cancer prevention. Such data might have provided further insight into the lifestyle choices that our subjects have made in their efforts to prevent breast and ovarian cancer, and cope with the increased stress related to knowing that they are at high risk of malignancy. Another limitation of the study is a relatively small sample size both overall and of cancer survivors, providing for minimal statistical power for some analyses. Finally, our study population should not be considered representative of all *BRCA *mutation carriers, since it was comprised of research volunteers who were almost exclusively white and highly-educated, both characteristics that are associated with higher rates of CAM use in the general population.

## Conclusion

We have documented that the rates of CAM use in our cohort of *BRCA*+ women, most of whom have never developed cancer, are roughly equivalent to the rates reported for women being treated for, or having survived, breast cancer, and seem to be higher than the rates reported for women from the general population. Further investigation will be required to better understand the intensity of, and motivations for, CAM use, as well as the relationships between CAM use, conventional screening practices, and cancer risk and worry in *BRCA+ *women. Data from a more representative sample of all mutation carriers would likely be very informative as well. However, given the high prevalence of CAM use in our subjects, especially the predominance of biologically-based therapies, which might pose a greater risk of unanticipated adverse effects, our study suggests that it is important for providers to inquire about, and to discuss the pros and cons of CAM use, with their *BRCA+ *patients [32].

## Abbreviations

Complementary and alternative medicine: CAM; National Center for Complementary and Alternative Medicine: NCCAM; National Health Interview Survey: NHIS; ductal carcinoma *in situ*: DCIS; Center for Epidemiologic Studies Depression: CES-D; transvaginal ultrasound: TVU.

## Competing interests

The authors declare that they have no competing interests.

## Authors' contributions

CMM, PLM, and JB performed the statistical analysis. CM was the primary author and wrote the final manuscript. All authors helped to conceive the study, participated in its design and coordination, and contributed to drafting the manuscript. All authors read and approved the final manuscript.

## Pre-publication history

The pre-publication history for this paper can be accessed here:


